# The Medical Register and Directory of the U. S.

**Published:** 1873-09-15

**Authors:** 


THE MEDICAL REGISTER AND DIRECTORY OF THE U. S.—If any of our readers have not answered the circulars sent them by Dr. S. W. Butler, asking for information needed to facilitate the correctness and speedy issuing of said Register and Directory, they had better do so without delay. The work is one of much importance, and every facility should be afforded the publisher to make it accurate in its details.

